# Proso Millet (*Panicum miliaceum* L.) as Alternative Source of Starch and Phenolic Compounds: A Study on Twenty-Five Worldwide Accessions

**DOI:** 10.3390/molecules28176339

**Published:** 2023-08-30

**Authors:** Diletta Balli, Maria Bellumori, Alberto Masoni, Michele Moretta, Enrico Palchetti, Bruno Bertaccini, Nadia Mulinacci, Marzia Innocenti

**Affiliations:** 1 Department of NEUROFARBA, University of Florence, Via Ugo Schiff 6, 50019 Florence, Italy; diletta.balli@unifi.it (D.B.); nadia.mulinacci@unifi.it (N.M.); marzia.innocenti@unifi.it (M.I.); 2 Department of Agriculture, Food, Environment and Forestry (DAGRI), University of Florence, 50144 Florence, Italy; alberto.masoni@unifi.it (A.M.); michele.moretta@unifi.it (M.M.); enrico.palchetti@unifi.it (E.P.); 3 Department of Statistics, Computer Science, Applications “G. Parenti”, University of Florence, Viale Morgagni 59, 50134 Florence, Italy; bruno.bertaccini@unifi.it

**Keywords:** antiradical activity, cinnamic acids, glycemic index, high amylose, resistant starch, starch composition

## Abstract

Proso millet has been proposed as an effective anti-diabetic food thanks to the combined action of polyphenols and starch. This study aimed to characterize the chemical composition of twenty-five accessions, in order to enhance this cereal as an alternative to available starch for food applications or to propose new food ingredients with health benefits. Proso millet contained a high percentage of starch, reaching values of 58.51%. The amylose content showed high variability, with values ranging from 1.36 to 42.70%, and significantly higher values were recorded for the white accessions than for the colored ones. High-resistant starch content (13.41–26.07%) was also found. The HPLC-MS analysis showed the same phenolic pattern in all the samples. Cinnamic acids are the most abundant compounds and significant differences in their total content were found (0.69 to 1.35 mg/g DW), while flavonoids were only detected in trace amounts. Statistical results showed significantly higher antiradical activity in the colored accessions than in the white ones.

## 1. Introduction

The rise in demand for gluten-free functional foods with low cholesterol and low glycemic index has required the use of alternative food sources. In this context, millet has attracted increased attention in the 21st century thanks to its flexibility towards climate changes, low water requirement and short production time [1,2,3]. Besides ecological benefits, millet has been extensively reviewed for its high health and nutraceutical values compared to other common cereals (e.g., rice, wheat, corn) [4]. In particular, regular consumption of millet has been reported to help in the management of type II diabetes by regulating glucose homeostasis [5,6]. This effect has been mainly attributed to millet’s carbohydrate quality and improved by the co-presence of phenolic compounds; indeed, the combination of polyphenols and starch makes millet an effective anti-diabetic food [7]. Starch-rich cereals are reported to contribute up to 50% of the total energy supply according to the World Health Organization (WHO) [8]. Demand for starch is never-ending and its industrial utilization depends on its availability; managing the shortage of cereals will be difficult for the starch industry due to global warming and climatic change, which is expected to occur in the future [8,9,10]. Thus, proso millet (*Panicum miliaceum* L.), included among the four major millets, will be a sustainable and environmentally friendly starch substitute for the manufacturing of resistant starch [11]. Proso millet starch typically accounts from 60% to 80%, with an amylose content ranging from 0.75% to 28.3% and a resistant starch content from 0.6% to 30% [12,13,14,15,16]. The consumption of resistant starch has been correlated with the reduction of postprandial glycemic and insulinemic responses, and this may have positive implications in the management of hyperglycemia and diabetes, considering that in a Western diet, this type of starch constitutes about 10% of the starch consumed.

The amylose content may be linked to millet’s hypoglycemic properties: the inverse relationship between amylose and glycaemic index is known, with studies showing that the addition of high amylose starch to diets modulates the glycemic response [17]. Lower enzymatic starch hydrolysis has been associated with (i) amylose’s lower surface area per molecule when compared to amylopectin, resulting in lower enzymatic binding and (ii) amylose’s higher susceptibility to retrogradation, thus resulting in a much lower rate of enzymatic attack [18,19]. 

Proso millet polyphenols, which mainly consist of hydroxybenzoic acids, hydroxycinnamic acids and flavonoids [20], are the other classes of molecules that may be exploited in the management of type 2 diabetes due to their inhibitory effects on starch digestive enzymes. Inhibitory effects of different classes of phenolic compounds on α-glucosidase and pancreatic α-amylase have been reported [21,22,23,24]. 

This study aimed to deepen the knowledge about proso millet, by conducting a chemical characterization of a world collection of twenty-five accessions. For this purpose, several analyses were carried out on grains: (i) the proximate analysis, (ii) the determination of total starch, resistant starch, digestible starch and amylose and amylopectin content, (iii) the determination of phenolic acids and flavonoids by HPLC-DAD-MS and (iv) the evaluation of antiradical activity by DPPH assay. In addition, a chemometric approach was applied to compare and possibly discriminate the millet samples according to their different characteristics. Greater knowledge of the nutritional and functional properties of proso millet varieties can contribute to proposing new food ingredients with potential health benefits.

## 2. Results and Discussion

### 2.1. Nutrient Composition

The protein, carbohydrate, sugar, fat, fiber, sodium and ash contents of the 25 millet accessions are reported in Table 1. Also, energy, expressed as Kcal, was determined. Carbohydrates represented the major macronutrient and the samples contained from 65.1 to 75.3 g/100 g total carbohydrates of which free sugars account for 0.6 to 1.8%. These values were in the same range of those previously reported in the study of Devisetti, et al. [25], in which the flour from proso millet whole grains showed a total carbohydrate content of 70 g/100 g.

In the review of Saleh et al. [4] the average nutritive value of different millet grains is reported; the carbohydrate content ranged from 55.0 to 72.6%, respectively, for barnyard millet and finger millet, while higher values are reported for other grains such as rice (76%) and maize (73%).

The second major nutrient was represented by proteins. Millet proteins, compared to other common cereals, are rich in essential amino acids such as leucine, alanine, methionine and cysteine. Indeed, as reported by Kalinova and Moudry [26], although the protein content of proso was similar to wheat, the grain of proso was significantly richer in essential amino acids (leucine, isoleucine, methionine) than wheat. Hence, the protein quality of proso (Essential Amino Acid Index) was higher (51%) compared to wheat. Table 1 reports values ranging from 8.70 g/100 g in SMS 3 to 14.00 g/100 g in SMS 660, similar to those found by previous literature studies [12,25,26]. In line with the literature [25], the fat content ranged from 2.6 to 5.1 g/100 g for most of the analyzed samples; only for four samples (SMS 3, SMS 106, SMS 132 and SMS 183) were higher values found. This may be due to the native differences of each variety, as showed by Zhang et al. [27] which reported a fat content ranged from 3.38 to 6.49% in 35 foxtail millets assessing that fat content is significantly affected by millet variety and cultivation area. Saturated fats accounted for about 10% of the total fats (Table 1). 

Concerning dietary fiber, an average content of 22.9% was observed. This value is similar to those already reported, although some samples showed higher values, probably due to the different genetic and agronomic parameters of each sample. Staple foods such as wheat and rice were reported to contain much lower levels of dietary fiber of around 8.0 to 9.0%, and this makes millet grains—and particularly proso millet—an important food crop.

The moisture content of grains is an important factor for shelf-life as low moisture content helps to enhance the storage of food grains. The moisture content of the 25 millets ranged from 8.8 to 10.9 g/100 g, in line with the previous studies. The mean ash content of the investigated millets is in the range of 2.6–7.8 g/100 g, which was significantly higher than the ash content observed in major millet sorghum (0.9–1.7 g/100 g) or in rice (1.3 g/100 g), wheat (1.6 g/100 g) and maize (1.2 g/100 g). The higher ash content of proso millet is indicative of its rich mineral content, as compared to other grains [4,28]. In this regard, proso millet is a good source of minerals like calcium, phosphorus, potassium, sodium, magnesium, manganese, iron, magnesium and zinc, as reported in the review of Das et al. [29]. Particularly, the average sodium content in our samples was 4.1 g/100 g. Proso millet is also important due to the fact that one-third of the energy in developing countries is derived from it; the average energy value for the studied accessions was 368 Kcal/100 g, in line with data reported in the literature [29]. 

**Figure 1:**
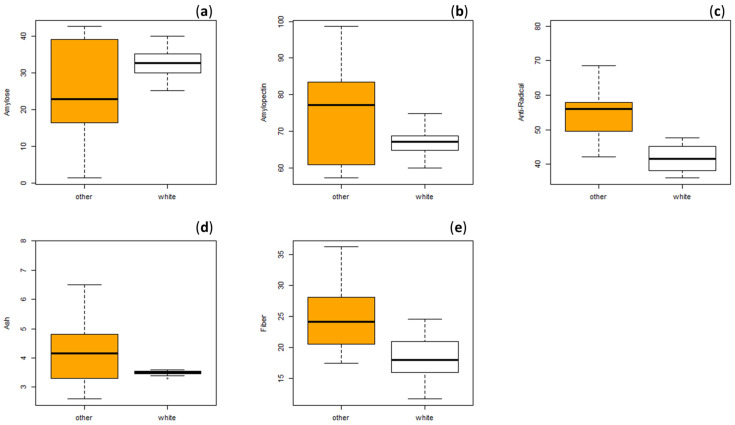
Box plot diagrams of (**a**) amylose, (**b**) amylopectin, (**c**) antiradical activity (%), (**d**) ash and (**e**) fiber contents (g/100 g) along with the white and colored millet samples.

As better discussed in Section 3.4, the 25 millet samples were grouped based on the color of the grains and subjected to statistical analysis in order to assess potential differences between colored and white species. Results showed significant differences in ash and fiber content between colored and white accessions. Particularly, the ash and the fiber content of colored millets was significantly higher (*p* < 0.05) than that of the white varieties (Figure 1d,e). This finding confirms what has already been reported in the literature on ash content in the study of Li et al. [30], in which the ash content of black millets was significantly higher compared to that of white varieties. As far as we know, no information about the fiber content based on the color of the variety was previously reported in the literature.

### 2.2. Starch Composition

Starch is the major component in millet and typically ranges from 56 to 65% of the total seed weight though up to about 80% starch has been reported for proso millet [17]. Amylose content of up to 34% was reported for foxtail, finger, proso and pearl millets and it may be linked to the hypoglycemic properties of these species. Indeed, the inverse relationship between amylose and glycemic index is known, with studies showing that the addition of high amylose starch to diets modulates glycemic response [17]. 

In this study, the amylose (A), amylopectin (AP), total starch (TS), resistant starch (RS) and digestible starch (DS) content in the twenty-five accessions of proso millet are evaluated and reported in Table 2.

The amylose content varied from 1.36 to 42.70 g/100 g, respectively, for samples SMS 675 and SMS 2, with a mean value of 27.20 ± 11.69 g/100 g. These results suggested a high diversity in amylose content among the 25 accessions, in line with previous literature data which reported amylose values ranging from 0 to 32.3% in 95 different proso varieties [1]. Our study showed diverse amylose types identified in our accessions: <5% of amylose content (2 accessions—waxy type); 5–20% (6 accessions); 20–30% (5 accessions) and > 30% (12 accessions), highlighting that the majority of samples were high amylose content type (A > 25%—non-waxy type).

The amylose content is related to the amylopectin one, as the latter was obtained by the difference of amylose (%) from 100. The amylopectin content in the 25 accessions ranged from 57.30 to 98.64 mg/100 g for samples SMS 2 and SMS 675, respectively. The average value was 72.70 ± 11.73 g/100 g, in line with previous literature data [16]. The variability in the amylose and amylopectin content was also observed in the study of Yang et al. [14], who reported values of amylose from 2.24% to 38.67%, respectively, in waxy and non-waxy varieties, and values of amylopectin from 25.44% (non-waxy) to 69.00% (waxy).

Different factors contribute to the hypoglycemic properties of millet and millet-based foods, such as the presence of proteins, lipids, α-amylase inhibitors, antinutrients and starch characteristics, as reported in the review of Annor et al. [17]. Particularly, the amylose/amylopectin ratio affects enzymatic starch hydrolysis kinetics since starches with higher amylose tend to have lower enzymatic starch hydrolysis rates. Indeed, amylose has a much lower surface area per molecule when compared to amylopectin resulting in lower enzymatic binding. Amylose chains are also more susceptible to retrogradation which results in changes in the conformation of the chains and thus results in a much lower rate of enzymatic attack. For these reasons, non-waxy starch of proso millet has many food applications compared to waxy starch [17,31]. In our study, an average total starch content of 46.95 g/100 g and an amylose/amylopectin ratio ranging from 0.01 to 0.75 was detected. Yang et al. [14] reported similar values and observed no significant differences in the total starch content between waxy and non-waxy types.

As explained in more detail in Section 3.4, statistical analysis applied to our samples showed that the amylose and amylopectin contents differed statistically differed (*p* < 0.05) according to variety color and, in particular, the white varieties contained higher amylose and lower amylopectin contents than the colored ones (Figure 1a,b). As far as we know, this point has not been previously investigated in millet grains. 

The starch that escapes enzymatic digestion in the small intestine and is passed to the large intestine where it undergoes fermentation is known as resistant starch. Literature reports that proso millets contain a highly resistant starch content and slowly digestible starch, particularly the non-waxy varieties [31]. In our accessions, resistant starch ranged from 13.41 to 26.07 g/100 g in SMS 132 and SMS 183 samples, respectively. These values are in line with those of Sharma and Gujral [12], who reported values of 21.99 ± 0.51 g/100 g for proso millet flours, higher than those of wheat flour (19.27 ± 0.93 g/100 g). Likewise, Yang et al. [14] reported values of resistant starch from 8.12 to 9.48% in waxy proso millet varieties, while higher values ranging from 16.56 to 19% in non-waxy varieties. In our accessions, no correlation between the amylose content (waxy and non-waxy varieties) and the RS content has been highlighted. Shen et al. [13] reported a lower RS content (from 0.64 to 2.21%) of nine proso millet varieties, again highlighting the non-waxy proso millet varieties significantly richer than waxy ones. 

Unlike the resistance starch, the digestible starch is hydrolyzed to glucose and absorbed by the small intestine. The starch digestibility of proso millet also depends on the variety and, in particular, the rapid digestible starch (RDS) generally ranges from 20.87% to 26.88%, while slow digestible starch (SDS) from 31.89% to 34.22 [8]. In our study, only the content of the total fraction of digestible starch was considered, without distinguishing between the two RDS and SDS. Results ranged from 20.49 g/100 g in samples SMS 132, SMS 656 and SMS 673 to 38.46 g/100 g in sample SMS 660 (Table 2), in agreement with the previously cited literature. Also, in this case, no correlation between waxy and non-waxy varieties and the DS content has been highlighted in our accessions, contrary to what was stated by Yang et al. [14].

A wide variation in starch composition was observed in our proso millet varieties. The information provided in this study should be valuable in guiding the selection of a suitable germplasm for breeding programs and food or non-food millet product development.

### 2.3. Phenolic Composition and Antiradical Activity

Until now, only a few kinds of metabolites of proso millet grains and brans have been investigated. Particularly, the recent study of Li et al. [32] performed a widely targeted metabolome analysis for four varieties of proso millet and provided a comprehensive metabolic profile of this crop. Among the identified compounds, only six phenolic acids were detected with significantly high levels, including ferulic acid, p-coumaric acid, vanillic acid, isoferulic acid, 4-hydroxybenzoic acid and p-hydroxycinnamic acid. In our study, the chromatographic profiles (Figure S1) presented the same phenolic pattern in all the twenty-five varieties and particularly, five cinnamic acids and two flavonoids were detected as shown in Table S1. The C-glycosylated flavonoid vitexin and isoferulic acid, methyl hydroxycinnamate and methyl ferulate were already reported in our previous works on millet [33]. 

Considering the extreme heterogeneity of the millet species, composition and phenolic content widely vary, depending on the type of millet grain. As reported in Table 3, the twenty-five accessions of millet considered in this study showed significant differences in total cinnamic acid content, with values ranging from 0.69 to 1.35 mg/g dried weight (DW) in SMS 198 and SMS 132 samples, respectively. This class of compounds was the most abundant, while flavonoids were not detectable in most of the samples, or present in very low amounts (0.01–0.10 mg/g). These results are in accordance with our previous works [33,34], in which millet samples from Burkina Faso (pearl millet, i.e., *Pennisetum glaucum* L.) showed a content of total phenols, extracted in the same acidic condition described in this manuscript, of 1.78 mg/g and 1.93 mg/g, respectively. Also, in the study of Devisetti et al. [25] similar values of 1.20 mg/g of phenolic content in the flour of proso millet whole grain were reported, with a total flavonoid content of 0.39 mg/g. As previously reported, the content of phenolic compounds and flavonoids varies with the type of millet and morphological fraction of the grain. In the review of Shahidi and Chandrasekara [35], the total phenolic content in proso millet was approximately 2.5 mg/g and flavonoids represented only 3% of the total phenolic fraction, while in other species (i.e., foxtail millet) flavonoids are present in a larger amount. Further, it has been shown that the content of phenolics differed depending on the variety and particularly, the brown varieties contained generally higher quantities than the white ones. Our results showed the highest phenolic content in the yellow-brown sample SMS 132, while the lowest effectiveness for the white variety SMS 198, although other white varieties have shown higher values.

Phenolic compounds, as natural antioxidants, improve the oxidative stability of foods and additionally have immense potential owing to their health-beneficial properties. In this study, the antioxidant properties of the twenty-five millets were evaluated in terms of DPPH radical scavenging capacity and displayed in Table 3. The antiradical activity varied from a minimum of 36% up to a maximum of about 79%, higher than those reported for proso millet in literature [12,32]. Particularly, sample SMS 3 showed the highest value, followed by SMS 191 (68.49%), SMS 668 (i.e., 62.60%) and SMS 185 (i.e., 61.50%). The sample SMS 198, characterized by the lowest total phenolic content, showed also the lowest level of antiradical activity (36.0%), followed by SMS 671 (37.20%), SMS 660 (38.95%) and SMS 183 (41.62%). A correlation of 0.39 (r, *p* < 0.05) was observed between the antiradical activity and the total phenolic content. Although in literature the significant contribution of phenolics to antioxidant activity was observed, a greater antiradical activity may depend not only on the phenolic content but also on several other factors, e.g., the chemical nature of the functional groups within the molecules, such as the position and number of hydroxyls, the binding energy and steric hindrance [22]. Sharma and Gujral [12] reported values of 29.85% of antioxidant activity for proso millet whole wheat flour, versus higher percentages found for finger (53.36%), foxtail (43.94%), barnyard (37.71%) and kodo (58.49%). Regarding other cereals, the same authors showed that millet flour possessed up to 78.54% higher antioxidant activity than whole wheat flour. Furthermore, a positive correlation of darker seed coat of grains with antioxidant activity was reported by the literature [12,36,37]. Our results confirmed this trend. Indeed, as also reported in the next paragraph, the antiradical activity showed statistically significant differences (*p* < 0.05) between white and coloured samples and, particularly, significant higher values were found for the coloured accessions (Figure 1c). In agreement with our results, DPPH radical scavenging activity of the phenolic fraction of proso millet reported in the study of Li et al. [32] ranged from 19.78 to 26.94% and the activity of the black variety was significantly higher than those of the other red, grey and white varieties, with white being the lowest.

### 2.4. Statistical Analysis

The combination of metabolite profiling or botanical characteristics with chemometrics has been widely used for food or crop products, in order to further direct breeding strategies for improving and optimizing the balance of food components. In this study, first, the Student’s *t*-test for the independent samples was applied to investigate the differences between the white and colored species of 25 millet samples, considering the agronomic characteristics (including number of leaves, weight of 1000 seeds, plant height, number of tillers, yield per plant, harvest yield and dry biomass), the nutritional and phenolic profiles and the antiradical activity. Table S2 shows the obtained results. Among the twenty-five considered parameters, only five showed statistically significant differences (*p* < 0.05) between white and colored accessions, particularly the amylose, amylopectin, fiber and ash contents along with the antiradical activity. Figure 1 reported the box plots of the five parameters for which a statistically significant difference was found. The white varieties showed a significantly higher average amylose content and a lower amylopectin content than the colored millet varieties. Furthermore, the colored varieties had significantly higher antioxidant capacity than the white ones, in accordance with other literature data [32,37]. Concerning the fiber and the ash amounts, the white varieties showed a significantly lower content than the colored ones.

The scatter plot matrix reporting the single values for the 25 millet species (Figure S2) showed no associations among the five parameters, except between amylose and amylopectin for which—as expected—a perfect association was observed.

**Figure 2:**
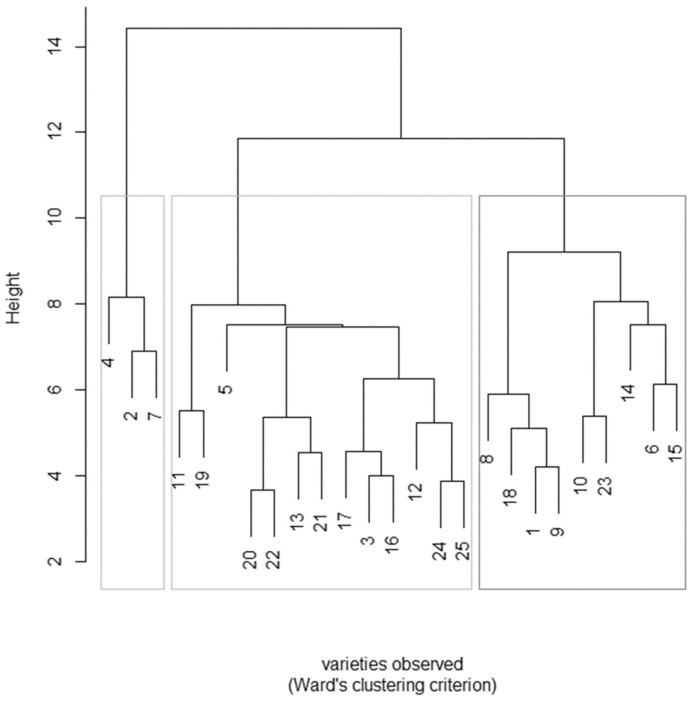
Dendrogram for the 25 proso millet samples obtained from the hierarchical cluster analysis. Euclidean distance and probability method between points. The numbers correspond to the list of samples in Table 4.

Furthermore, to inspect the existence of millet species with similar properties a hierarchical cluster analysis (HCA) was conducted on all parameters. The relative dendrogram is reported in Figure 2.

HCA evaluates data according to their homogeneity and heterogeneity, grouping them according to the degree of similarity. The results were obtained using Ward’s method in order to obtain the minimum variance between the vectors that comprise each group, besides Euclidean distances, to verify the similarities between samples. The multielement evaluation classified the species into three major clusters, comprising, respectively, 3, 13 and 9 individuals, confirming the high variability among the individual varieties. Indeed, the 25 accessions were selected among 80 on the basis of their heterogeneity of geographical origin and seed color. The samples contained in Cluster 1 (SMS 4, SMS 2 and SMS 7) were characterized by the highest values of energy (Kcal and Kj) and the highest content of fats, while for the accessions of the other groups, no common parameters were found.

## 3. Materials and Methods

### 3.1. Chemicals

All solvents were of analytical HPLC grade from Sigma Aldrich (St. Louis, MO, USA). Ultrapure water was obtained by the Milli-Q-system (Millipore SA, Molsheim, France). Sodium hydroxide (≥98%), sulfuric acid (95.0–98.0%), ferulic acid, vitexin and vitexin-2-*O*-rhamnoside standards were purchased from Sigma Aldrich (St. Louis, MO, USA). 

### 3.2. Samples

Twenty-five proso millet samples (*Panicum miliaceum* L.) were selected from a world germplasm collection of 80 accessions, belonging to the United States Department of Agriculture (USDA), according to their agronomic performances [38] and considering the high variability in seed colors and geographical origin. Millet accessions were cultivated in a field trial during the spring-summer 2019, in the Tenuta di Cesa (experimental farm of Tuscan Regional Administration) located in Cesa (Italy, 43°18′32″ N; 11°49′35″ E). At the farm, the climate was typically Mediterranean with a monthly mean temperature of 21 °C and a cumulative rainfall of 378 mm for the whole proso millet growth period (May-Aug, data collected with a local weather station). The soil was characterized by a clay texture (25.4% sand; 30.1% silt; 44.5% clay), a pH of 7.1, low electrical conductivity (EC; 0.154 mS cm^−1^), high cation exchange capacity (CEC; 27.46 meq 100 g^−1^) and by an organic matter content of 1.66%. The previous crop in the experimental field was wheat (*Triticum aestivum*). The seedbed was prepared using a disc harrow (20–25 cm depth) in winter, followed by a spike-tooth harrow (6–8 cm depth) before the seeding operation. No fertilization was applied, and no irrigation was administered. Each proso millet variety was sown on a 25 m^2^ plot (all arranged on the same field), the 10 of May and they were harvested the 30 August 2019. Plots were manually weeded as required, during the crop growth, without the use of pesticides. At harvest time, grains were collected from each plot, cleaned and stored at 10 °C for the following analyses. Codes, plant names, origin and agronomic parameters of the analyzed millet samples are reported in Table 4.

**Table 4:** Code, plant name, origin and agronomic parameters of the analyzed millets (*Panicum miliaceum* L.). NR, not reported.

	Code	Plant Name	Nation	Color	Number of Leaves	Weight of 1000 Seeds (g)	Plant Height (cm)	Number of Tillers	Yield per Plant (g)	Harvest Yield (Kg ha^−1^)	Dry Biomass (Kg ha^−1^)
1	SMS 2	GE.2013-28	Georgia	green	5.8	5	69.5	4.7	5.33	956.1	3391.9
2	SMS 3	Index Semnium 295	France	brown	7.8	5.08	69.2	4.3	8.38	1365.8	4741.5
3	SMS 27	Kumdari Beyaz	Turkey	white	6.8	4.93	64.8	5.2	11.17	1877.2	6620.5
4	SMS 106	Arzen	Afghanistan	yellow	7.2	5.53	53.3	3.3	13.85	1723.9	5836.4
5	SMS 132	IPM 1092	Iran	yellow/brown	8.3	5.53	77.8	4	11.4	2441.8	8383.8
6	SMS 174	Crown	Canada	green	4.2	4.85	35.7	3.2	7.4	970.5	2999.8
7	SMS 183	Kharkov 25	Ukraine	white	6.7	6.78	47.5	4.7	6.5	1154.2	3982.9
8	SMS 185	NR	Ukraine	yellow/brown	4.8	6.53	61.7	4.8	14.43	1436.2	4886.9
9	SMS 189	NR	Australia	yellow/brown	5.3	5.65	76.7	5.3	9.22	1323.1	4389.4
10	SMS 191	NR	Australia	green	5.3	5.22	66.8	4.7	6.5	2544.5	7052.7
11	SMS 198	White French Strn. 8567-7	Australia	white	7.3	4.9	80.8	4.2	10.63	2318.9	8078.3
12	SMS 202	NR	Nepal	yellow	8.3	5.95	84	3.3	6.68	2075.7	6999.5
13	SMS 208	Vishenutu	Taiwan	yellow	7.8	6.52	99.7	2.7	6.6	1279.1	3906.3
14	SMS 209	Lung Shu no. 5	China	red	4.7	5.83	85.7	3.7	10.98	2496.2	7478.5
15	SMS 211	Lung Shu no. 14	China	black	5.8	5	69.5	4.7	5.33	956.1	3391.9
16	SMS 648	GR 658	Marocco	white	7.8	5.08	69.2	4.3	8.38	1365.8	4741.5
17	SMS 655	Domace Biele	Czechoslovakia	yellow/brown	6.8	4.93	64.8	5.2	11.17	1877.2	6620.5
18	SMS 656	Dunakiliti “A”	Hungary	red	8	4.97	70.5	4.8	4.65	1298.5	4329.1
19	SMS 660	Harkovskoe 2	Germany	white	7.2	6.92	75.5	2.5	12.55	2894.9	9154.3
20	SMS 668	Malcaltor “A”	Hungary	red	7.3	6.48	69.3	2.8	8.4	1727.8	5710.9
21	SMS 671	Prosos	Kenya	white	6.7	5.97	60.5	3.2	5.68	1354.1	4576.3
22	SMS 673	Saratovskoe 953	Russia	green	6	6.88	61.3	3.8	11.27	1407.6	5177.5
23	SMS 675	Strzeleckie brown	Poland	black	5.8	4.42	64.7	4.3	9.4	2291.9	8127.1
24	SMS 679	Tojdenskoe 215	Russia	red	6.2	5.85	89.2	4.7	8.3	2163.8	7728.1
25	SMS 700	Minco	USA	white	8	5.85	66.8	3.5	5.57	1928.5	6660.7

### 3.3. Proximate Analysis

The millet flours were analyzed for protein, fat, ash and moisture content according to ISS protocol (1996/34). A Soxhlet extraction was used to gravimetrically determine the fat content, the protein content was determined by the Kjeldal method, with N × 6.25 (N = total nitrogen). Dietary fiber was assessed according to AOAC Method 985.29. Total carbohydrate content was calculated as a difference, using the following formula: Carbohydrates (%) = 100 − (% crude protein + % total ash + % crude fat). The total energy content was expressed in calories, using the conversion factors 4.0 for crude proteins, 9.0 for crude fat, and 4.0 for carbohydrates. The analytical values were evaluated from the mean of three determinations for each sample.

### 3.4. Determination of Resistant, Digestible and Total Starch

Resistant starch and digestible starch were determined using the K-RAPRS 11/18 Megazyme Resistant Starch Assay Kit (Megazyme International Ireland Ltd., Wicklow, Ireland), based on both AOAC method 2002.02 and AACC method 32–40. The procedure employed a mixture of pancreatic α-amylase and amyloglucosidase to hydrolyze digestible starch to D-glucose; the hydrolysis was carried out for 4 h at 37 °C. The reaction was terminated by the addition of ethanol and the resistant starch was recovered as a pellet on centrifugation. This was then washed twice by suspension in ethanol (50% *v*/*v*), followed by centrifugation. The free liquid was removed by decantation. Resistant starch was dissolved in 1.7 M NaOH by stirring in an ice-water bath and this solution was then neutralized with acetate buffer and the starch was hydrolyzed to D-glucose with amyloglucosidase. D-glucose was measured with glucose oxidase/peroxidase reagent (GODOP) and this was the measure of the resistant starch content in the sample. Digestible starch was determined by pooling the original supernatant and the washings, adjusting the volume to 100 mL and measuring D-glucose content with GODOP reagent. Absorbance was measured at 510 nm using an Agilent 8453 G1103A spectrophotometer (Agilent Technologies). Resistance starch and digestible starch (g/100 g) were calculated as the amount of glucose × 0.9. 162/180 = 0.9 factor to convert from free D-glucose, as determined, to anhydro-D-glucose, as occurs in starch. Total starch was calculated as the sum of digestible and resistant starch. 

Amylose and amylopectin were determined using the K-AMYL 06/18 kit (Megazyme International Ireland Ltd., Wicklow, Ireland), employing a procedure based on the specific precipitation of amylopectin by concanavalin-A lectin (Con A). Ground samples (25 mg) were dispersed by heating in 1 mL of dimethyl sulfoxide (DMSO). Lipids were removed by precipitating the starch in ethanol and recovering the precipitated starch. After the dissolution of the precipitated sample in an acetate/salt solution, amylopectin was specifically precipitated by the addition of Con A and removed by centrifugation. The amylose, in an aliquot of the supernatant, was hydrolyzed to D-glucose, which was analyzed using GODOP reagent. The total starch, in a separate aliquot of the acetate/salt solution, was similarly hydrolyzed to D-glucose and measured colorimetrically by glucose oxidase/peroxidase. The concentration of amylose in the starch sample was estimated as the ratio of GOPOD absorbance at 510 nm of the supernatant of the Con A precipitated sample to that of the total starch sample.

Each sample was analyzed in triplicate.

### 3.5. Extraction and Determination of Phenolic Compounds by HPLC-DAD-MS Analyses

The extraction of phenolic compounds was performed according to Balli et al. [33]. Briefly, 1 g of defatted flour was extracted with 25 mL of MeOH/H_2_SO_4_ (1.2 M *v*/*v*) with the aid of an ultrasonic bath (180 °C for 55 min). The obtained extracts were then centrifuged (13,148× *g* for 10 min) and analyzed by HPLC-DAD-MS. 

All the extracts were analyzed using a HP 1260L liquid chromatography equipped with a DAD detector (Agilent Technologies, Palo Alto, CA, USA). The identification of phenolic compounds was performed by an HP 1260 MSD single quadrupole mass spectrometer with an API/electrospray interface (Agilent Technologies) by comparing their retention times, UV–Vis and MS spectra with those of the respective standards (in the case of luteolin-7-*O*-glucoside, vitexin and isoferulic acid) or with our previous published data [33]. A Raptor column ARC-18 (150 × 3 mm, 5 μm, Restek S.r.l., Cernusco sul Naviglio, MI, Italy) was used. The elution method started from 100% of solvent A (H_2_O^+^ pH 3.2) and 0% of solvent B (CH_3_CN) as reported by Balli et al. [33]. The UV–Vis spectra ranged from 200 nm to 500 nm and the chromatograms were acquired at 330 and 350 nm. Total time of analysis 42 min, flow rate 0.8 mL/min and injection volume 10 µL. MS operating conditions were as follows: N_2_ flow of 10.5 L/min; gas temperature of 350 °C; capillary voltage of 3500 V; nebulizer pressure of 1811 Torr. The acquisition of the spectra was performed in negative ionization, with a fragmentation energy of 80 eV.

Phenolic acids were quantified using a five-point calibration curve with ferulic acid as external standard (purity ≥ 99%) at 330 nm, linearity range of 0.00–0.41 µg (R^2^ = 1.0). Flavonoids were quantified using a five-point calibration curve with vitexin (purity ≥ 99%) at 350 nm, linearity range of 0.00–1.23 µg (R^2^ = 1.0).

### 3.6. DPPH Radical Scavenging Activity

The antiradical activity was evaluated by using the stable radical DPPH (1,1-diphenyl-2-picrylhydrazyl) test, as described in Ieri et al. [39]. In detail, millet phenolic extracts were added in a 1:1 amount to an ethanolic solution of DPPH (0.04 mg/mL). Measurements were carried out at 517 nm with a DAD 8453 spectrophotometer (Agilent Technologies) at time 0 and every 2 min for the following 30 min. Antiradical activity (AR%) was calculated using the relationship: [AR% = 100 × (A_0_ − A_30_)/A_0_], where A_0_ and A_30_ were the absorbance of DPPH, respectively, at time 0 and after 30 min from the addition of the extract.

### 3.7. Statistical Analysis

Each measurement experiment was performed in triplicate and results were expressed as mean ± standard deviation. Firstly, samples were grouped in order to investigate differences between white and colored millet species, considering the agronomic characteristics, the nutritional and phenolic profiles and the antiradical activity. In particular, to determine whether the means of the two groups of millet species differed we conducted an independent sample *t*-test, applying the Welch correction to the degrees of freedom if the two groups have unequal variances [40]. 

Due to the small sample set, we intentionally did not perform a multiple comparison correction. As is well known, this class of corrections has been introduced in the literature to reduce the chance of false discovery in case of multiple comparisons. It is almost certain that the introduction of a multiple comparison correction would lead to the acceptance of the null hypothesis in all the tests performed since those techniques are almost always conservative. But we have to argue that when sample sets are small, tests are not very powerful and the risk of false acceptance of the null hypothesis is very high. The introduction of any conservative technique with the aim of reducing the chance of false discovery will lead to an increase in the probability of a false negative (type II error).

Therefore, a Hierarchical Cluster Analysis (HCA) was conducted on all parameters. To inspect the existence of millet varieties with similar properties. HCA is a multivariate statistical method (frequently used in data mining) that allows verifying the existence of natural groupings inside a set of available observations (identified by the values of some measured features). Groups are formed iteratively, by successively joining or splitting groups of observations. We follow the agglomerative strategy in which each observation starts in its own single group, and groups are successively paired until forming the largest possible one identified by the whole set of available observations. Observation closeness was calculated on the basis of the Euclidean distance, and the Ward hierarchical agglomerative method was used as grouping criteria in order to obtain the minimum variance between the observations that comprise each group.

Prior to this chemometric approach, the variables were scaled (transformed with zero mean and unit variance) to standardize the statistical importance of all the measured features. 

All the statistical analyses were conducted using R (version 4.2.1).

## 4. Conclusions

This study aimed to deepen the knowledge about proso millet, by conducting a chemical characterization of a world collection of twenty-five accessions. Due to its nutritional qualities, proso millet proves to be an important food crop. Indeed, this cereal contains a high percentage of starch, reaching values of 58.51 g/100 g in our accessions. The amylose content showed high diversity among the varieties and the majority of samples were high amylose content type (non-waxy type), particularly interesting since its link to the hypoglycemic properties and the inverse relationship between amylose content and glycemic index. Proso millets showed high-resistant starch content, ranging from 13.41 to 26.07 g/100 g, higher as compared to wheat flour values reported in the literature. The starch digestibility of proso millet also depends on the variety; digestible starch varied from 20.49 to 38.46 g/100 g in our accessions.

Concerning the phenolic profile, the HPLC-MS analysis showed the presence of the same phenolic pattern in all the samples, while the phenolic content widely varies. Significant differences in total cinnamic acid content were found, ranging from 0.69 to 1.35 mg/g; flavonoids were only detected in trace amounts. DPPH radical scavenging capacity varied from a minimum of 36% up to a maximum of 79%, and the statistical results highlighted a correlation between antiradical activity and the color of grains; particularly, colored accessions showed significantly higher antiradical activity compared to white ones. Similarly, statistical analysis showed that the white varieties contained a higher amylose content and lower amylopectin content than the colored ones. As far as we know, this is the first time that this result has been reported for millet grains. The wide variation in the chemical composition observed in our samples should be valuable in guiding the selection of suitable germplasms for breeding programs and food or non-food millet product development. Furthermore, due to the promising functional properties of proso millet starch responsible for the eating quality and cooking parameters of millet, this study could be useful to propose proso millet as an alternative to commercially available starch for food applications.

## Figures and Tables

**Table 1:** Proximate composition of proso millet samples. The values are a mean of triplicates; the relative standard deviation was below 5%.

Code	Kcal/100 g	Ash	Moisture	Proteins	Carbohydrates	Sugars	Fats	Saturated	Na	Fibre
		g/100 g	g/100 g	g/100 g	g/100 g	g/100 g	g/100 g	g/100 g	mg/100 g	g/100 g
SMS 2	367	3.8	9.1	8.9	74.4	0.6	3.8	0.4	4.7	19.9
SMS 3	398	3.2	9.6	8.7	68.8	0.6	9.7	1	3.9	17.5
SMS 27	365	3.3	10.7	10.9	70.7	1	4.4	0.4	4.1	18
SMS 106	389	3.7	10.1	12.4	65.1	0.8	8.8	0.9	3.5	36.3
SMS 132	376	4.2	10.9	12.1	65.6	0.9	7.2	0.7	6.8	28.4
SMS 174	357	6.5	10	11.9	68.4	0.8	4	0.4	8	20.7
SMS 183	394	3.5	10.2	10.9	65.5	1.3	9.8	1	1	16.6
SMS 185	362	4.7	9.4	8.9	73.4	1.3	3.6	0.4	0.8	21.2
SMS 189	359	4.5	9.3	10.2	73.1	1.2	2.9	0.3	1.1	19.3
SMS 191	369	4.8	9	11.4	69.9	1.2	4.9	0.5	1.2	25.5
SMS 198	372	3.6	9.8	12.1	69.4	1.7	5.1	0.5	7.5	11.7
SMS 202	354	6.3	10	11.7	68.3	1.2	3.8	0.4	9	23.8
SMS 208	372	2.8	10.3	12.5	69.7	1.1	4.8	0.4	0.5	22.5
SMS 209	352	7.8	8.8	12.3	67.4	1.6	3.7	0.5	9.7	29.9
SMS 211	365	3.3	9.7	13.5	70.2	1.6	3.4	0.3	8.7	33.4
SMS 648	362	3.5	10.5	11.9	70.7	1.2	3.4	0.4	7.7	24.6
SMS 655	360	4.1	9.6	13.2	70.1	1	2.9	0.3	1.7	28.1
SMS 656	372	2.6	8.9	9.4	75.3	1.1	3.7	0.4	0.4	24.6
SMS 660	369	3.4	9.5	14	69	1.4	4.1	0.4	2.7	22.1
SMS 668	369	4	9.7	13.1	68.6	1.5	4.7	0.4	1.1	27.9
SMS 671	366	3.5	10.4	13.8	68	1.5	4.3	0.4	1.1	19.9
SMS 673	368	4.3	9.5	11.5	70.2	1.5	4.5	0.5	2.2	26.5
SMS 675	369	2.9	9.4	10.9	73	1.3	3.7	0.4	3.9	20
SMS 679	357	5.2	10.2	12	69	1.8	3.6	0.4	2	20.6
SMS 700	357	4.3	9.6	12.9	70.7	1.7	2.6	0.3	1.5	15.4

**Table 2:** Amylose (A), amylopectin (AP), resistant starch (RS), digestible starch (DS) and total starch (TS) of the 25 millet samples. Data are expressed as g/100 g as mean of triplicates ± standard deviation.

Samples	A (g/100 g)	AP (g/100 g)	A/AP	RS (g/100 g)	DS (g/100 g)	TS (g/100 g)
SMS 2	42.70 ± 3.18	57.3	0.75	14.12 ± 3.03	30.53 ± 2.33	44.65
SMS 3	26.74 ± 2.36	73.26	0.37	16.53 ± 0.00	36.58 ± 0.17	53.11
SMS 27	40.06 ± 3.15	59.94	0.67	19.07 ± 1.98	35.57 ± 3.23	54.64
SMS 106	3.61 ± 0.52	96.39	0.04	19.11 ± 1.08	36.49 ± 0.26	55.6
SMS 132	40.41 ± 4.08	59.59	0.68	13.41 ± 1.12	20.49 ± 1.66	33.9
SMS 174	17.03 ± 0.89	82.97	0.21	19.15 ± 1.71	25.76 ± 2.62	44.91
SMS 183	34.72 ± 2.88	65.28	0.53	26.07 ± 1.66	32.44 ± 1.95	58.51
SMS 185	19.74 ± 1.95	80.26	0.25	14.91 ± 1.55	35.15 ± 1.76	50.06
SMS 189	32.81 ± 5.83	67.19	0.49	15.74 ± 0.55	21.98 ± 1.09	37.72
SMS 191	17.76 ± 0.01	82.24	0.22	22.57 ± 0.03	24.09 ± 0.61	46.66
SMS 198	29.87 ± 2.81	70.13	0.43	18.95 ± 2.38	29.69 ± 1.30	48.64
SMS 202	24.04 ± 2.44	75.96	0.32	18.62 ± 0.44	30.72 ± 3.56	49.34
SMS 208	21.57 ± 1.24	78.43	0.28	19.93 ± 3.62	24.04 ± 1.21	43.97
SMS 209	16.46 ± 2.11	83.54	0.2	14.26 ± 0.65	21.40 ± 0.29	35.66
SMS 211	14.25 ± 1.48	85.75	0.17	16.52 ± 1.81	32.38 ± 1.18	48.9
SMS 648	32.74 ± 2.98	67.26	0.49	19.93 ± 1.73	22.98 ± 5.67	42.91
SMS 655	39.97 ± 2.96	60.03	0.67	20.14 ± 1.67	31.86 ± 1.76	52
SMS 656	16.46 ± 2.11	83.54	0.2	17.55 ± 0.12	20.49 ± 3.41	38.04
SMS 660	35.64 ± 5.30	64.36	0.55	14.54 ± 1.01	38.46 ± 2.41	53
SMS 668	39.76 ± 1.46	60.24	0.66	15.92 ± 1.08	28.90 ± 0.07	44.82
SMS 671	25.21 ± 2.47	74.79	0.34	18.62 ± 0.44	30.72 ± 3.56	49.34
SMS 673	37.95 ± 0.08	62.05	0.61	18.18 ± 4.13	20.49 ± 3.41	38.67
SMS 675	1.36 ± 0.04	98.64	0.01	16.62 ± 1.77	31.56 ± 1.56	48.18
SMS 679	39.16 ± 4.35	60.84	0.64	24.74 ± 6.06	25.87 ± 2.31	50.61
SMS 700	30.09 ± 4.77	67.91	0.45	20.25 ± 1.10	29.56 ± 3.08	49.81

**Table 3:** Total cinnamic acid content (TC), total flavonoid content (TF) and antiradical activity of millet samples. Data are expressed in mg/g DW and as percentages as mean of triplicates ± standard deviation. nd, not detected.

Samples	TC (mg/g DW)	TF (mg/g DW)	Anti-Radical Activity (%)
SMS 2	1.05 ± 0.04	nd	55.42 ± 1.13
SMS 3	1.21 ± 0.03	nd	79.93 ± 1.61
SMS 27	1.08 ± 0.01	0.02 ± 0.01	43.69 ± 2.65
SMS 106	0.90 ± 0.08	0.09 ± 0.01	56.56 ± 0.91
SMS 132	1.35 ± 0.05	0.01 ± 0.01	57.63 ± 2.39
SMS 174	1.13 ± 0.01	nd	49.50 ± 0.87
SMS 183	1.01 ± 0.12	nd	41.62 ± 1.14
SMS 185	1.11 ± 0.02	nd	61.50 ± 3.24
SMS 189	1.25 ± 0.01	nd	50.81 ± 4.71
SMS 191	1.18 ± 0.1	0.03 ± 0.00	68.49 ± 0.99
SMS 198	0.69 ± 0.01	nd	36.00 ± 0.09
SMS 202	1.25 ± 0.18	0.05 ± 0.00	51.11 ± 0.98
SMS 208	1.11 ± 0.01	nd	55.70 ± 1.78
SMS 209	1.18 ± 0.14	nd	43.14 ± 0.60
SMS 211	1.33 ± 0.07	0.02 ± 0.00	57.84 ± 1.42
SMS 648	1.17 ± 0.21	nd	46.67 ± 1.66
SMS 655	1.47 ± 0.09	nd	48.82 ± 2.22
SMS 656	1.16 ± 0.03	nd	56.47 ± 0.08
SMS 660	0.93 ± 0.01	nd	38.95 ± 2.25
SMS 668	1.17 ± 0.04	nd	62.60 ± 1.03
SMS 671	0.95 ± 0.03	nd	37.20 ± 1.42
SMS 673	1.18 ± 0.02	nd	42.90 ± 2.30
SMS 675	0.98 ± 0.05	0.10 ± 0.00	57.36 ± 2.16
SMS 679	1.17 ± 0.05	nd	42.09 ± 3.36
SMS 700	1.28 ± 0.01	nd	47.70 ± 2.73

## Data Availability

Data sharing not applicable.

## Supplementary Materials

The following supporting information can be downloaded at: https://www.mdpi.com/article/10.3390/molecules28176339/s1, Figure S1. HPLC profiles at 330 and 350 nm of millet extract. The identified phenolic compounds are listed in Table S1; Figure S2: 2D scatter plot matrix of amylose, amylopectin, fiber and ash contents and antiradical activity measured on the 25 millet samples. Colored samples are shown in orange; the white varieties are represented with blue circled white bullet; Table S1: Identified compounds in millet samples according to UV–Vis (sh, shoulder) and mass spectra; Table S2: Independent sample T test conducted on white and colored millet species. * Represent *p* value < 0.05. G1, mean value for colored millet species; G2, mean value for white millet species; df, degree of freedom; A, amylose; AP, amylopectin; RS, resistant starch; DS, digestible starch; TC, total cinnamic acid content; TF, total flavonoid content; ARA, antiradical activity.
