# Supplementary material for: Proso Millet (Panicum miliaceum L.) as Alternative Source of Starch and Phenolic Compounds: A Study on Twenty-Five Worldwide Accessions
Source: Molecules. 2023 Aug 30;28(17):6339. doi: 10.3390/molecules28176339 (PMC10489065; doi:10.3390/molecules28176339)
Supplement: Supplementary file 1 [file molecules-28-06339-s001.zip › molecules-2519510-supplementary.pdf]

SUPPLEMENTARY MATERIAL

Table S1. Identified compounds in millet samples according to UV–Vis (sh, shoulder) and mass spectra.

Phenolic compounds	Rt	λ max	MW	$[M\text{-}H]^-$	Identified compounds
**F1**	11.6	270,349	448	447	Luteolin-7-*O*-glucoside
**F2**	13.3	268,336	432	431	Vitexin
**C1**	15.1	287sh,323	194	193	Isoferulic acid
**C2**	18.8	290sh,310	178	177	Methyl hydroxycinnamate
**C3**	19.7	300sh,324	208	207	Methyl ferulate
**C4**	24.0	288sh,324	240	239	Cinnamic der.
**C5**	30.9	306sh,324	414	413	Cinnamic der.

Rt, retention time

Table S2. Independent sample T test conducted on white and coloured millet species. * Represent *p* value < 0.05. G1, mean value for coloured millet species; G2, mean value for white millet species; *df*, degree of freedom; A, amylose; AP, amylopectin; RS, resistant starch; DS, digestible starch; TC, total cinnamic acid content; TF, total flavonoid content; ARA, antiradical activity.

	**G1: coloured**	**G2: white**	**t-test**	***df***	***p*-value**	
A	25.10	32.62	-2.12	23	0.045	*
AP	74.90	67.05	2.22	23	0.036	*
RS	17.50	19.63	-1.49	9	0.170	
DS	27.71	31.35	-1.59	13	0.138	
TC	1.18	1.02	2.06	8	0.072	
TF	0.02	0.00	1.73	21	0.098	
ARA	55.44	41.69	4.92	21	0.000	*
Kcal	367.50	369.29	-0.34	11	0.742	
Kj	1556.56	1563.29	-0.32	11	0.755	
Ash	4.37	3.59	2.26	21	0.035	*
Moisture	9.64	10.10	-2.11	13	0.056	
Proteins	11.37	12.36	-1.65	13	0.122	
Carbohydrates	70.03	69.14	0.87	16	0.383	
Sugars	1.17	1.40	-1.79	15	0.094	
Fats	4.65	4.81	-0.17	9	0.872	
Saturated fats	0.48	0.49	-0.08	9	0.938	
Sodium	3.84	3.66	0.14	12	0.891	
Fibre	24.78	18.33	3.17	13	0.007	*
Number of leaves	6.41	7.21	-2.18	23	0.040	
Weight of 1000 seeds	5.57	5.78	-0.58	9	0.578	
Plant height	70.52	66.44	0.77	15	0.452	
Number of tillers	4.13	3.94	0.46	10	0.654	
Yield per plant	8.85	8.64	0.17	11	0.872	
Harvest yield	1685.32	1841.94	-0.59	10	0.569	
Dry biomass	5619.54	6259.21	-0.77	10	0.462	

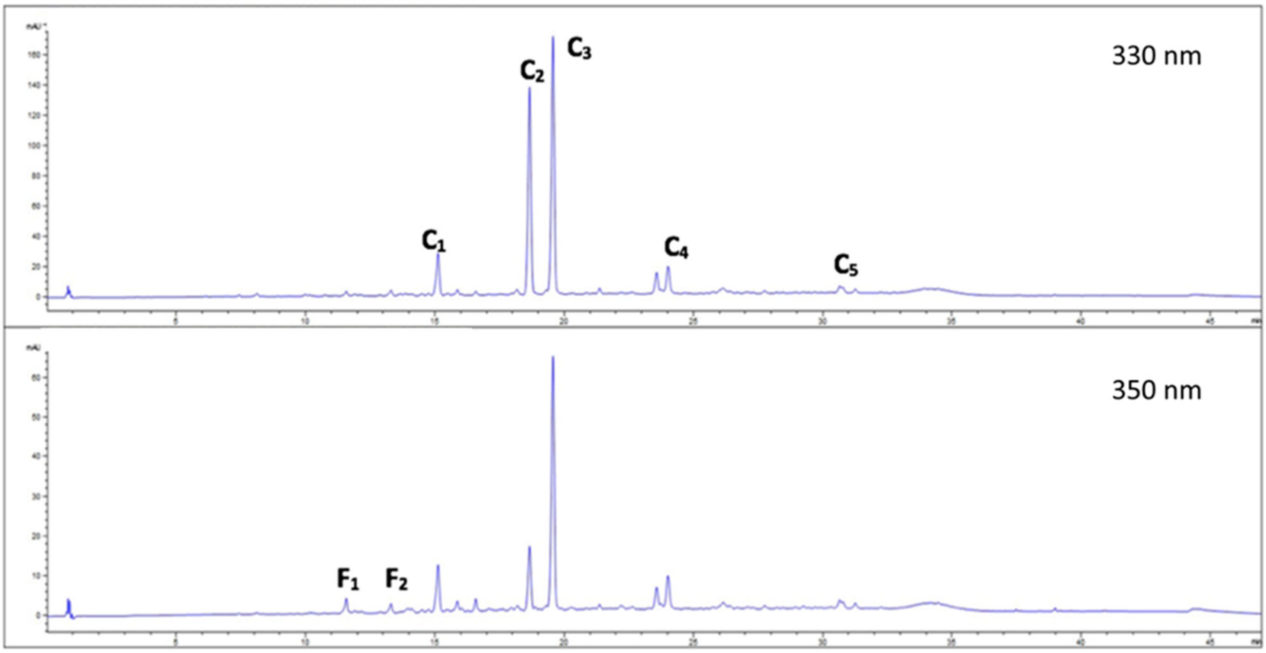

Figure S1. HPLC profiles at 330 and 350 nm of a millet extract. The identified phenolic compounds are listed in Table S1.

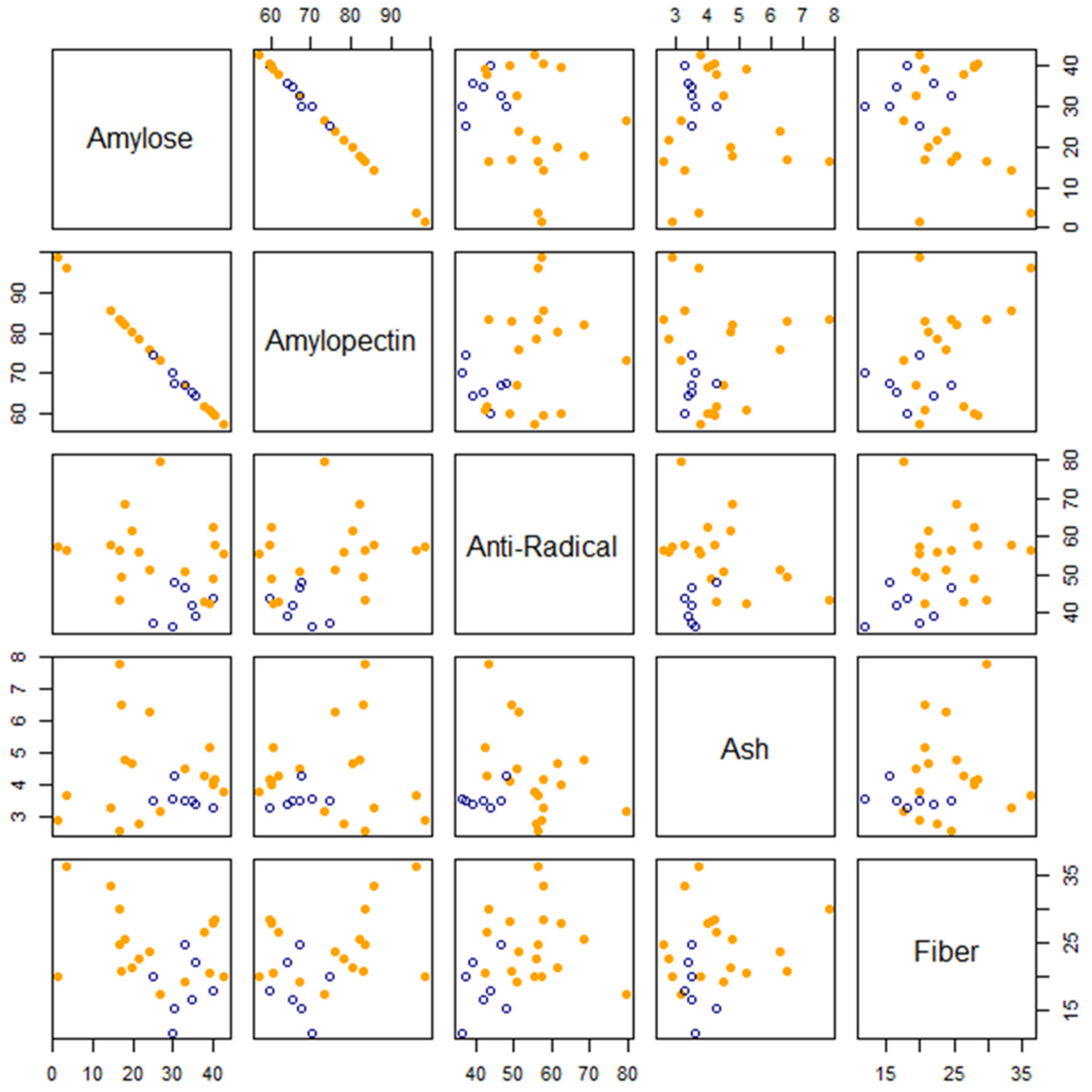

Figure S2. 2D scatter plot matrix of amylose, amylopectin, fibre and ash contents and antiradical activity measured on the 25 millet samples. Coloured samples are shown in orange; the white varieties are represented with blue circled white bullet.
